# Design Issues in Personalized Nutrition Advice Systems

**DOI:** 10.2196/37667

**Published:** 2023-03-29

**Authors:** Iris M de Hoogh, Machiel J Reinders, Esmée L Doets, Femke P M Hoevenaars, Jan L Top

**Affiliations:** 1 Research Group Microbiology & Systems Biology Netherlands Organization for Applied Scientific Research Leiden Netherlands; 2 Department of Endocrinology Leiden University Medical Center Leiden Netherlands; 3 Wageningen Economic Research Wageningen University & Research Den Haag Netherlands; 4 Wageningen Food & Biobased Research Wageningen University & Research Wageningen Netherlands

**Keywords:** personalized nutrition, eHealth design, health measurements, dietary advice, behavior change support, knowledge rules, modeling, sense, reason, act

## Abstract

The current health status of the general public can substantially benefit from a healthy diet. Using a personalized approach to initiate healthy dietary behavior seems to be a promising strategy, as individuals differ in terms of health status, subsequent dietary needs, and their desired behavior change support. However, providing personalized advice to a wide audience over a long period is very labor-intensive. This bottleneck can possibly be overcome by digitalizing the process of creating and providing personalized advice. An increasing number of personalized advice systems for different purposes is becoming available in the market, ranging from systems providing advice about just a single parameter to very complex systems that include many variables characterizing each individual situation. Scientific background is often lacking in these systems. In designing a personalized nutrition advice system, many design questions need to be answered, ranging from the required input parameters and accurate measurement methods (sense), type of modeling techniques to be used (reason), and modality in which the personalized advice is provided (act). We have addressed these topics in this viewpoint paper, and we have demonstrated the feasibility of setting up an infrastructure for providing personalized dietary advice based on the experience of 2 practical applications in a real-life setting.

## Introduction

### Background

Sufficient physical activity and a healthy diet are known to substantially contribute to the prevention of chronic diseases and obesity [1-4]. The increasing burden of chronic diseases, including diabetes, cardiovascular diseases, and obesity, highlights the importance of promoting a healthy lifestyle [5-7]. Dietary and physical activity guidelines have been developed for the general population with the aim to prevent or delay the onset of chronic diseases. However, only a limited number of people adhere to the general guidelines for healthy dietary intake and physical activity [8-12]. In addition, these general guidelines are mostly *one size fits all* and disregard differences between individuals in terms of biology, behavior, genetics, and the sociopsychological environment [13,14]. Therefore, it has been hypothesized that personalized approaches may be more effective in changing lifestyle parameters such as diet, sleep, stress, and physical activity [15]. Here, we focus on nutrition.

### Personalized Nutrition

The overall goal of personalized nutrition approaches is to promote or maintain health status of an individual by using personal data to tailor dietary recommendations or services to fit their specific needs [16]. Personalized nutrition has been defined as approaches that “use individual-specific information, founded in evidence-based science, to promote dietary behavior change that may result in measurable health benefits” [17]. Studies have shown that such personalized approaches are more effective in improving dietary behavior as compared with generic information [18-24]. These personalized nutrition approaches can use the knowledge that individuals may show a differential physiological response to nutrients, foods, or dietary patterns [25,26] but may also be primarily focused on individual (dietary) behavior, preferences, and goals [16,18]. Several factors could explain the high effectiveness of a personalized approach. First, the advice itself is tailored to the individual’s constitution and preferences and therefore may be expected to be more effective than generic advice; in other words, if personal data are used for generating evidence-based dietary recommendations, they are more likely to result in health benefits as compared with dietary recommendations based on population data [13,27]. Second, information that is perceived as more relevant by an individual is more likely to receive attention, thereby increasing the impact of the information and the feeling of involvement of an individual [27,28].

### Personalized Nutrition Advice Systems

It becomes apparent from the variety of studies performed so far [26,29-32] that personalized advice covers many ways of personalization, ranging from personalization based on a single nutrient (eg, salt intake and sodium status) to complex systems including a multitude of measurements and associated recommendations. In addition, the mode of delivery for personalized nutrition may vary, including personalized coaching by a dietician, personalized meal services, and personalized recommender systems such as apps or platforms or combinations thereof [33]. In this paper, we focused on the development and implementation of digital personalized nutrition advice systems (PNASs), in which the translation of individual data into a personalized service is digitalized and (partly) automated. Such PNASs contain 3 common components:

- *Sensing part,* which is made up of the input parameters that are used, such as biomarkers, behavioral data, or genetic information
- *Reasoning part,* which translates the input parameters into an advice using knowledge rules such as scientifically substantiated food-health relations
- *Acting part,* where the advice is communicated to the consumer with the aim to help them move toward more healthy habits [16,34]

This is also consistent with Berezowska et al [35], who state that PNASs typically consist of three information process stages forming a feedback loop, describing the relation between a service providing personalized dietary advice (eg, provider of a dietary advice app) and the user of that service: (1) an individual user first provides their personal information, (2) this information is processed to obtain a personalized advice, and (3) it is then communicated to the user of the service. It is clear that during the development of a digital personalized advice system, a multitude of design choices need to be made on these 3 levels. A schematic overview of a PNAS, including possible measurement platforms for the sensing part, possible modalities for the acting part, and examples of potential target groups is provided by Adams et al [17].

### Development of a PNAS and Application in 2 Use Cases

In this paper, we have described the rationale behind the design choices made in developing our PNAS. This PNAS was developed as part of the Public Private Partnership Personalized Nutrition and Health, in which the Netherlands Organisation for Applied Scientific Research and Wageningen University and Research work together with private parties [36]. We aimed at developing a science-based, dynamic PNAS that could be used in different target groups. To test the PNAS, 2 use cases were defined, one aiming at consumers with premetabolic syndrome motivated to change their diets to enhance their health (ie, highly motivated consumers; n=37) and the other aiming at consumers with a low socioeconomic status (n=96). The PNAS had to have a generic backbone but be able to handle different sets of input and output parameters. A digitalized PNAS that was suitable for use in these 2 use cases was developed and tested using 2 human studies [37,38].

This paper discusses the 3 generic components in PNASs—sensing, reasoning, and acting in general—and the design choices that were made for sensing, reasoning, and acting for our PNAS in the 2 use cases. First, we expand on the possible input parameters that can be used for sensing, including their caveats. Regarding reasoning, we will discuss how evidence-based food-health relations can be incorporated into a digital system and about the modeling strategies that we used. Regarding acting, the focus is on the communication of feedback (health and dietary status) and advice (dietary recommendations) and the use of behavior change techniques for activating consumers.

## Sensing—Personal Data

### Overview

When the main goal and target group of the PNAS are clear, the first step in the designing process is to decide on input or *sensing* parameters that can and should be included. There is a wide variety of possible parameters, including clinical measures, behavior, well-being, genetics, personal preferences, and so on [15]. Broadly, 2 categories of relevant data for personalization can be distinguished, namely, biological characteristics and data related to current behavior, preferences, barriers, and objectives [16]. To decide which sensing parameters to include in your PNAS, decisions need to be made about the scope of your system, for example, what aspects of health are relevant; which measurements are feasible; and what parameters need to be included to measure the success of your PNAS, for instance, in improving the health status or well-being of consumers [17].

For our PNAS, we decided to include both biological characteristics or *health parameters* and parameters related to current lifestyle behavior, which are further described in the following sections.

### Health Parameters

To decide which health parameters to include in your system, it is important to consider what aspects of health are influenced by nutrition and how these can be measured. The concept of health used to be defined as a state of complete mental, physical, and social well-being [39]. However, currently, a more holistic approach is accepted, in which there is also a role for an individual’s ability to cope with daily challenges [40]. The physical dimension of health, represented by the body and its overall functionality, covers a broad scope ranging from the absence of disease to the level of physical fitness. Classical physical health in a pharmacological or clinical setting is determined by the *phenotype*. The phenotype is often assessed by benchmarking anthropometric measurements and single overnight fasting plasma biomarkers against cutoff values that represent either a *healthy* or *compromised* status. Currently, health is considered as the body’s ability to adapt to (changing) circumstances, while remaining within homeostatic boundaries [40]. This definition calls for alternative assessment methods that operationalize health by capturing the body’s systemic response to (challenging) circumstances, instead of a single measurement such as fasting glucose [41]. When only clinical cutoffs are considered, subtle changes in overall health status may be overlooked. Therefore, the integration of multiple measured biomarkers or phenotypic traits is key. For this purpose, a so-called health space model can be used, which combines multiple biomarkers into a single score using multivariate statistical methods and data from reference populations [42,43].

The first step for incorporating health in a PNAS is to determine which phenotypic health parameters provide a good representation of the health status of a person and may provide the opportunity for personalized advice. In the simplest sense, parameters such as sex, age, and anthropometrics can be used, but often, more specific and clinical measurements (ie, plasma biochemical markers and vitals) and genotypic data are included to provide a more holistic overview of health. All measurements should be accurate, valid, and preferably relatively easy to obtain.

In our system, basic characteristics such as age and sex were combined with easy-to-measure anthropometrics and vitals such as body weight, blood pressure, body length, and waist circumference. Depending on the target population and the setting of the investigation, the set of measurements was either extended or reduced. One of our studies was performed with highly motivated consumers in a health care setting. Therefore, participants could be easily called to the clinic, which allowed for more extensive anthropometric measurements (eg, fat percentage) and the collection of blood samples for measuring plasma biomarkers (high-density lipoprotein [HDL] cholesterol, low-density lipoprotein [LDL] cholesterol, glucose, and triglycerides and a nutrient profile). The other study with consumers with low socioeconomic status was set in a supermarket, which asks for easy-to-perform measurements with direct readouts. In such a setting, do-it-yourself measurements can be applied, such as body weight scales, blood pressure meters, and handheld devices for plasma biomarkers. In addition, wearables and apps could provide a good solution for assessment in a more do-it-yourself setting but were not part of our studies. The measurements to be included in a PNAS are also dependent on the target group. Highly motivated individuals were more willing to accept blood sampling [37] to be able to receive more detailed personal advice, as compared with those with low socioeconomic status who were less interested in nutrition-related solutions for health [38].

### Lifestyle Behavior Parameters

In principle, a complete set of health parameters only, with sufficiently accurate values, could suffice in providing a personalized advice. However, in addition to the physical dimension of health, lifestyle, mental, and socioenvironmental factors influence health status, for example, food intake, physical exercise, stress, physical environment, and social support networks. Knowledge about an individual’s current lifestyle will have added value in several ways. First, the set of health parameters is not sufficiently rich to cover all required aspects. Second, relations between lifestyle behavior and health status are not fully known, and this for instance limits the number of nutrients, foods, or food groups for which recommendations can be formulated [13]. Providing personalized recommendations relative to current food intake behavior can help to fill these gaps and enrich the personalized advice; however, the recommendations themselves will be based on population-based cutoff values. Third, to some extent, current lifestyle behavior can also serve as a proxy for health status [44]. Finally, integrating current lifestyle behavior into personalized advice could increase the relevance of recommendations to match individuals’ current behavior, and their habits and preferences, for instance, current physical activity patterns, can be incorporated [18]. Overall, two main approaches can be distinguished for the measurement of lifestyle behavior, namely (1) monitoring current lifestyle and (2) self-reporting on lifestyle behavior [45,46]. Monitoring can be performed using sensors and mobile apps, such as an activity tracker or a sleep monitor. Self-reporting mostly relies on questionnaires, which vary in the level of obtained detail and validity.

In our system, in terms of lifestyle, we focused on dietary intake. For measuring dietary intake over a period, several self-reporting measurement methods are available, including (1) food frequency questionnaires, which are commonly used to provide a quantitative measure of nutrient level; (2) food diaries or dietary recalls, which provide a quantitative output on nutrient level and product level; and (3) healthy eating indexes, which provide a more qualitative output in terms of overall healthiness of the diet [47-49]. However, these conventional tools for measuring dietary behavior have major limitations. They are subject to overreporting and underreporting, dependent on the willingness of a user to log their dietary intake and the quality and details of the underlying food composition databases [47-49]. As an alternative approach, nutrient profiling in blood could be considered to capture dietary intake. This would include plasma levels of specific vitamins and minerals or metabolites as a proxy for dietary status [50,51]. In addition, a few biomarkers that can reflect dietary intake on a food-group level exist, for example, alkylresorcinols for whole grain intake, lipid profiling for fish intake, and a combination of carotenoids and vitamins for fruit and vegetable intake [52,53]. Unfortunately, most of these biomarker levels reflect long-term dietary behavior rather than short-term food intake; in contrast, many metabolites have a short half-life and thus are not representative of habitual intake [50]. In addition, currently, biomarkers do not provide a complete picture of an individual’s dietary intake. However, combining biomarkers with more *classical approaches* such as food diaries and questionnaires may result in a more accurate assessment of dietary intake [27]. Another approach to assess dietary intake is to use a diet quality index to assess adherence to a specific dietary pattern, such as the Healthy Eating Index and the Mediterranean Diet Adherence Screener [54,55]. Such diet quality indexes are based on population-based dietary guidelines and provide insight into the overall diet quality and conformance with recommended intakes of specific food groups that are key to a healthy diet. In addition, combinations of dietary assessment methods can be used.

As our studies were set in the Netherlands, for our system, we used the Dutch Healthy Eating Index (DHEI) questionnaire. The DHEI includes a set of 15 categories or *food groups*, consists of a relatively simple list of questions that can be answered in a short time frame, and results in a score ranging from 0 to 10 for the 15 food groups and an overall diet quality score [56,57]. The reason for using this dietary assessment method is that we are not very interested in obtaining the exact intake of macronutrients and micronutrients by individuals but merely in whether individuals comply with dietary guidelines at a food-group level as a first step toward personalization. Using food groups for personalized advice instead of nutrients has several advantages: (1) focusing on food groups is more consistent with the shift in the Dutch dietary guidelines from single nutrients to food groups [58]; (2) both evidence-based relations between food groups and health parameters and between nutrients and health parameters can be incorporated in the system, using specific nutrients to further fine-tune the advice [59,60]; and (3) feedback and advice are easily applicable as consumers do not have to identify which foods are high in the recommended nutrients themselves. The disadvantage of using DHEI is that no information on the consumption of specific food products or the amount of macronutrients and micronutrients consumed can be obtained [57]. In addition, the DHEI provides output on a qualitative level, not quantitative; for instance, the DHEI does not provide insight into the exact amount of vegetables consumed (in grams). The DHEI is based on population-based dietary guidelines and can be used for distinguishing between categories ranging from healthy to unhealthy dietary behaviors for the included food groups. Such a subdivision can be used for personalizing dietary recommendations based on current dietary intake patterns. To further personalize the recommendations, this output can be combined with other parameters, such as health status markers, as described in the previous paragraph, which we applied in our PNAS.

## Reasoning—Advice Model

### Overview

From the abovementioned arguments, it follows that 3 basic design choices have to be made when developing digital systems for personal dietary advice:

- First, the health condition of an individual should be used as the actual variable to control. Health parameters are monitored, and their values are used to decide which aspect of dietary intake should be focused on [16,27]. This requires a model that maps health parameter values to nutrients, foods, or food categories that need attention. This model should be based on science-based relations between dietary intake and health impact [17].
- Second, current individual food intake should be considered as a starting point for an individual path toward healthy intake, rather than directly pointing to the optimal diet. Eating patterns cannot be changed at will; habits are difficult to change [61]. Therefore, we do not assess products in terms of *healthy or not healthy for a representative population* but analyze eating habits in terms of deficiencies or gaps with respect to the ideal situation [62]. For our system, we decided that measuring food intake at the category level was sufficient.
- Third, in principle, personal preference, motivation, and situation have to be taken into account to ensure compliance with the generated advice [18]. In this study, this was limited to asking consumers to self-select which food category to focus on and to formulate implementation intentions for these, with the latter being a behavior change technique facilitating actual behavior change (refer to the *Behavior Change Techniques* section).

These design choices give rise to 2 questions. How can we create a software-based model that generates a personal advice, and how can we combine food intake data with health data in this model? In the following sections, we address the modeling approaches that we used for our PNAS, knowledge acquisition as a basis for an expert-driven model, and composing personalized advice in a flexible manner.

### Modeling Approach

For creating a model that links the observed health variables (such as blood pressure, LDL cholesterol level, and BMI) to food categories that need attention, ideally, we start from scientific publications and reports. However, constructing software models from scientific papers is a complicated and time-consuming process. In practice, we assume that dietary professionals already have operationalized this knowledge. For our system, we have applied knowledge acquisition methods from information science to develop the required software models. We consulted nutrition experts to informally describe the heuristics they use in practice in the form of, for example, a text file or a spreadsheet. They also provide pointers to the underlying scientific evidence. This process of *mining* expert knowledge [63] results in many knowledge rules. These rules can be expressed as decision trees or, more flexibly, as logical knowledge rules. However, for our system, we have selected Bayesian Belief Networks (BBNs) to express the relation between the observed health parameters and suggested changes in diet (ie, advice).

BBN is a probabilistic model that represents a set of variables and their conditional dependencies using a directed graph. The advantage of this approach is that tools are available to start with an initial qualitative influence diagram and then progressively add quantitative details. The experts can then evaluate the overall effect of the combined heuristics they provided by running the reasoning capabilities of the network and verify the predictions made.

In our case, the network represents the probabilistic relationships between health variables (input) and advice variables (output). Each of the parameters in our model has several discrete values (states); for example, for the input parameter systolic blood pressure, we used the values *90 to 110*, *110 to 129*, and *130 to 250*. Each state in the outputs refers to a text fragment that is provided to the consumer, for example, “no_advice,” “high-blood-pressure_eat_sufficient_vegetables,” and “enrich_with_nuts.” The links between the input and output parameters express the probability that the state of one parameter leads to a certain state of the other parameter, expressed as percentage. In our current model, we assume that if each of the input parameters is in a single state (ie, this state has a probability of 100% and the other has 0%), then each of the output parameters is also in a single state. However, the strength of BBNs is that they permit a distribution of probabilities over multiple states, allowing uncertainty in the inputs and outputs. For example, if BMI is unknown and the probability of high waist circumference is 45%, there is a 10% chance that no advice is needed regarding vegetable intake. However, determining these probability values manually is very difficult and considered as future research.

An important advantage of the BBN is the fact that it is possible to combine explicit expert heuristics with additional observational data as knowledge sources to compute probability values or even create the network itself. Once we have collected enough data from observations regarding how dietary advice parameters have influenced the health parameters, it is possible to *train* the model with these data and automatically adapt the probability tables. In this study, the amount of data collected was not sufficient to perform this next step.

### Knowledge Acquisition

The process of knowledge acquisition involves several rounds of interviews with dietary experts and systematic record keeping, in which domain experts and knowledge engineers work together. Such a process often reveals hidden assumptions, differences in terminology, and controversies among experts. Overall, 2 methods can be used to resolve such issues: *confrontation among experts* and *relating to underlying science.*

A typical issue in knowledge acquisition that often raises debate among experts arises from conflicting classifications. For example, the general Dutch guidelines use the category, *bread*, for a range of products and provide cutoff values for healthy consumption. However, some food intake apps use the category, *whole grain product*, for different types of products such as bread, pasta, and rice. How should we then classify *whole grain bread*? This type of semantic discussion among experts can often be resolved by applying appropriate modeling practices. In this case, *bread* can be used as a *class,* defining a group of products that use similar ingredients and preparation methods. In contrast, the concept, *whole grain*, indicates that a product is produced from whole grains, which should be expressed by the *property*, “is_produced_from,” rather than by defining a distinct *class*, *whole grain products*. In this way, *whole grain bread* is a type of *bread*, with property, “is_produced_from = whole_grain.” Another interesting but confusing category is the notion of *unhealthy product*, in particular, in the context of personalized advice. By assigning food products as instances of this predefined class, we assume that these products are unhealthy for anyone in any situation. This contradicts with the idea that what is healthy depends on the individual, their entire dietary pattern, and their context and should therefore be modeled as a (derived) *property* rather than a *class*.

For the second approach to solving knowledge conflicts, that is, *relating to underlying science*, the experts were asked to select relevant publications to support the heuristics they expressed. This is certainly not a straightforward task. In food-health research, scientific studies often use *nutritional values* as input variables, as these basic metabolic and physiologic mechanisms are often known. However, relations expressing the impact of entire foods and dietary patterns on health are also needed, as these take nutrients in a food matrix and interactions between foods into account. These 2 different approaches (nutritional values vs food categories and diets) also need to be reflected in the knowledge rules that are used in individual advice. In our study, we have chosen to stay at the level of food categories and have the experts decide on how nutrient-level evidence is incorporated. For example, for some categories, we know that they are typically high in a particular nutrient (eg, omega-3 fatty acids in fish). In this case, we can use the evidence at the nutrient level for the associated food category. In addition, we have used nutrient-health relations to elaborate the final advisory texts. For example, we recommend types of fruit that are high in fiber for people with high blood pressure, as fiber can help to reduce blood pressure.

Finally, the experts also needed to decide whether the conditions under which the underlying studies were originally performed can be generalized to our system and target groups. As our system is developed for use in the Netherlands, for instance, evidence from meta-analysis on food-health relations in the Asian population was excluded [64]. In addition, the level of evidence and quality of the research should be considered, for instance, using the Grading of Recommendations, Assessment, Development, and Evaluations method [65]. Regarding our knowledge rules, we used evidence as given by the Dutch Health Council, which applies a thorough but time-consuming literature review process to translate observed health effects in cohort studies to generalized rules for the impact of food categories on health, as a basis. We extended this basic set with rules derived from more recent literature and additional publications covering health parameters that were not within the scope of the Dutch Health Council (eg, glucose, triglycerides, and HDL cholesterol levels and waist circumference). An example of the latter is a meta-analysis showing the beneficial effects of whole grain consumption on blood glucose levels and risk of type 2 diabetes [66-68]. The additional knowledge rules that were added to our system also include food-health relations as accepted for health claims by the European Food Safety Authority, based on evidence from a recent meta-analysis.

### Composing the Advice

In this section, we explain in more detail how we algorithmically combined health parameters and food intake measurements to create a composite personal advice.

First, the input nodes of BBN were defined as the amount of intake for the categories used in the DHEI questionnaire (ie, vegetables, fruit, whole grain products, salt, fish, dairy, sugar-containing beverages, butter, nuts, coffee, alcohol, and unhealthy snacks) and the values of several health parameters (ie, BMI, waist circumference, diastolic and systolic blood pressure, glucose, triglycerides, LDL, HDL, carotenoids, alkylresorcinols, and omega-3 fatty acids index). Second, the output nodes for the network were then broken down into separate advice texts at 4 *levels* (Textbox 1).

**Figure 1:**
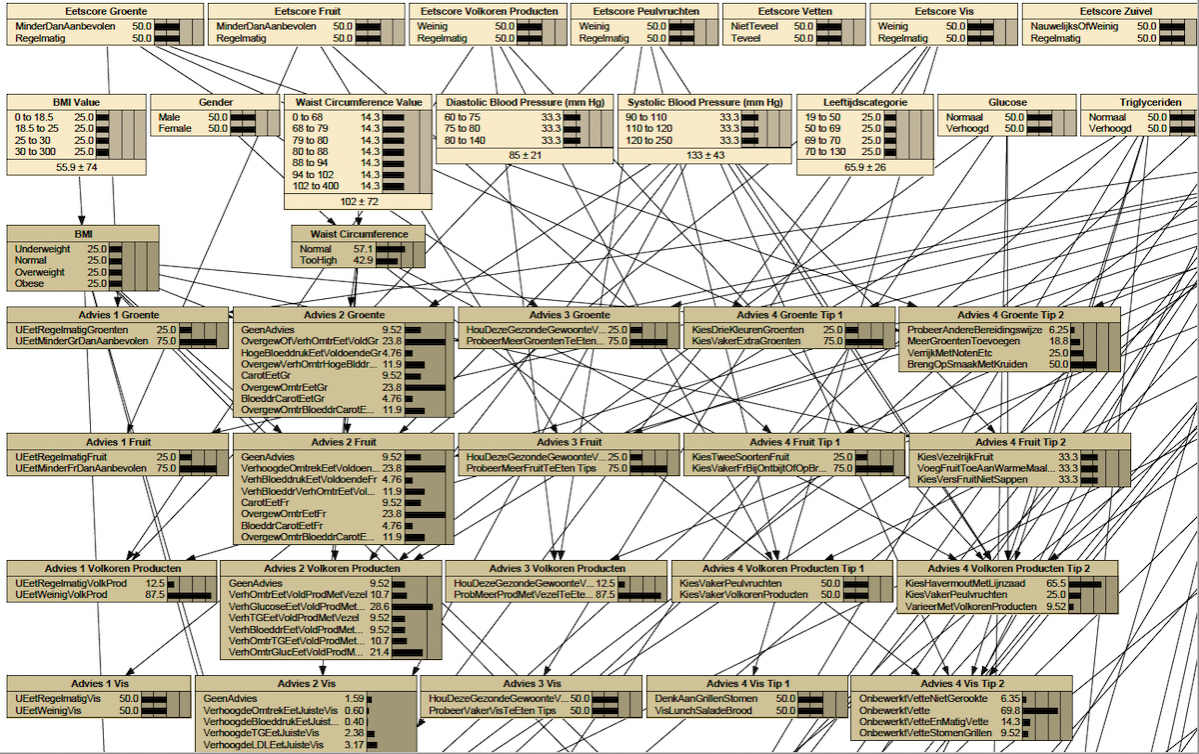
Part of the Bayesian Belief Network. The first and second rows contain input values. The first row contains intake values for the food categories from the Dutch Healthy Eating Index questionnaire. The second row contains values for the included health measurements. The third row includes some intermediate parameters, that is, BMI and waist circumference (qualified values). The lower rows show the 4 levels of advice, each row presenting a food category. Note that all values are divided into discrete ranges.

Then, BBN connects the input nodes to the output nodes, following the heuristics provided by the food experts. This is illustrated in Figure 1. In several iterations, we adjusted the transition probabilities between the nodes to verify and improve the impact of the collective input values on the outcome of the model. In this way, we achieved consensus among the experts based on their professional knowledge and experience.

**The 4 advice levels in our personalized nutrition advice system.**

**Advice levels**

1. The first level provides feedback about the current food intake—does a consumer comply with the dietary guidelines for a food group? For example, the advice text could state “You eat little fish.”
2. The second level indicates whether the consumer’s health status directs attention to a specific food category. For example, “Because of your high blood glucose level it is very important for you to consume sufficient dietary fiber.”
3. The third level motivates consumers to increase, maintain, or decrease their current dietary intake, based on the output of the first 2 levels. For example, “Try to eat more fiber-rich products.”
4. The fourth level provides both general and personalized practical tips. The first tip is linked to the current dietary intake and provides the suggestion to increase or decrease current intake (if not meeting the guidelines) or to increase variation (if meeting the guidelines). The second tip contains practical advice for a consumer to improve their health status. For example, “Choose oatmeal for breakfast, and add a full tablespoon of flaxseed to add extra fiber.”

## Act—Communication for Behavior Change

### Overview

The final step in the process of providing personalized advice is communicating the personalized advice to the consumer. The way in which the advice is provided to the consumer is critical, as studies have shown that individuals pay more attention to recommendations that are perceived as more relevant to them [69]. Several approaches can be used to increase perceived relevance of the advice by the consumer. In our PNAS and studies, the following 4 approaches for personalized communication were applied:

- Provide feedback about consumer’s current lifestyle and health status to confront them with their actual behavior or health status, motivate them to take action, and monitor their improvements over time
- Increase the relevance and applicability of the advice itself by ensuring that it fits a consumer’s preferences, for example, by adjusting the framing and format of the advice to the personal characteristics of the receiver
- Determine an appropriate source—the source of the advice plays an important role in determining the credibility of the advice
- Apply behavior change techniques to increase the involvement and compliance of the consumer with the advice, for instance, by using implementation intentions or if-then plans specifying when, where, and how one will achieve a certain behavior [70].

These 4 approaches are further discussed in the following sections.

### Feedback About Health Status and Behavior

In both studies in which we applied our PNAS, we not only provided personalized advice to the participants but also feedback about their current health and dietary status. In the study with highly motivated consumers, feedback and advice were provided via a web-based portal and discussed during a telephone consultation with a dietician. Follow-up was performed via email. In the study with consumers with low socioeconomic status, the feedback and advice were provided via a report that was sent to participants via email. As consumers should not be overwhelmed by the feedback, long lists of data that consumers cannot interpret or do not know how to act on should be avoided. To make feedback about health status more easily understandable by consumers, several strategies can be applied, such as color coding or composite scores that give an overall view of the health status or benchmarking personal data to the general population or peer groups (for an example, refer to the study by Morrow et al [71]).

**Figure 2:**
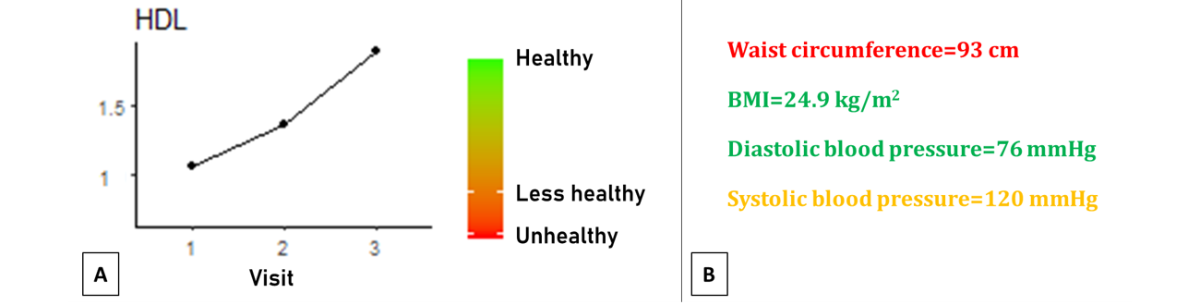
Examples of color-coded feedback about individual biomarker values. (A) Graphical representation as used in the study with highly motivated consumers; (B) textual representation as used in the study with consumers with low socioeconomic status. HDL: high-density lipoprotein.

In our PNAS, we provided consumers with their individual scores on relatively well-known single biomarker values (eg, blood pressure, body weight, and cholesterol) and plotted them against generally accepted healthy ranges. Color coding was used to indicate whether the individual values were within the healthy range, borderline, or outside the healthy range (Figure 2). This strategy has been shown to increase understanding of health data, especially for people with low numeracy skills [72].

**Figure 3:**
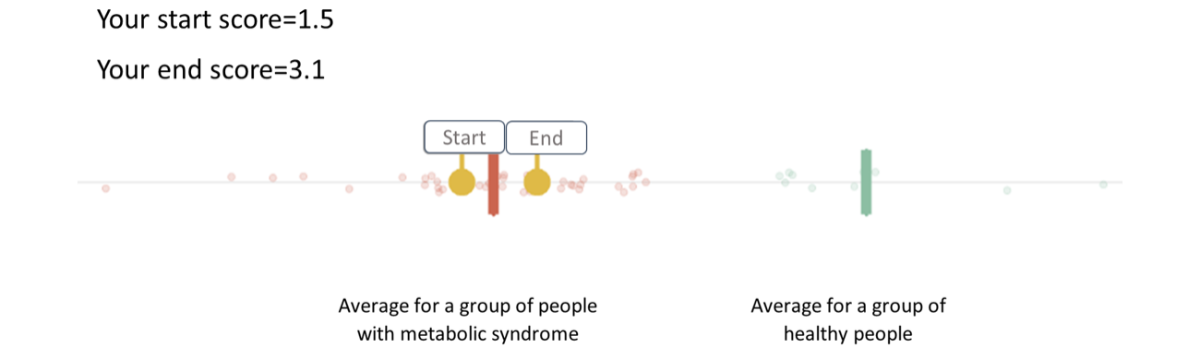
Example of composite score for overall metabolic health, as provided to participants in the study with highly motivated consumers.

In addition, in the study with highly motivated consumers, we provided health status feedback by integrating the outcomes of all markers into a composite score for overall *metabolic health* (Figure 3). To calculate the metabolic health score, a so-called health space model was developed (refer to the *Sensing—Personalized Data* section). Visualizations of such a metabolic health score may provide a valuable tool in communicating overall health status to individuals and can be used to show how an individual scores as compared with peers or to monitor changes in health over time [15,73]. This may make it easy for individuals to see their progress over time, as compared with having to weigh changes in various health parameters themselves.

Furthermore, in the study with consumers with low socioeconomic status, feedback about dietary intake was provided by means of stars per food category (eg, vegetables, whole grains, and unhealthy snacks), which reflected a score on a scale from 1 to 10 (Multimedia Appendix 1). Visualizing these scores helped to improve understanding in a group with low socioeconomic status [38]. Nevertheless, although we tried to make it simple, especially in the study with consumers with low socioeconomic status, some individuals indicated in the evaluation of the study that the feedback was sometimes difficult to understand (Figure 2 and Multimedia Appendix 1). This outcome indicates how difficult it is to provide the correct format of feedback and how concisely these aspects have to be adjusted to the target group. As our studies were performed with 2 different target groups and the provided feedback and advice formats were not identical between these studies, no firm conclusions can be drawn on how to best differentiate in communicating feedback between these target groups. Future studies including various target groups that are exposed to various types of feedback and advice should be performed to further investigate how feedback can best be communicated to (different types of) consumers.

### Relevance and Applicability of Personalized Advice

Motivation to receive personalized advice and engagement in a personalized dietary program are essential for the success of a PNAS. To increase this motivation, personalized recommendations should connect to the most important goals that consumers may have regarding receiving personalized dietary advice. The results of a consumer survey conducted within the Personalized Nutrition and Health program revealed that having a clear health goal (eg, losing weight) is a more important determinant of success than *just obtaining insight* into one’s dietary pattern and obtaining possibilities for improvement [74]. In addition, a difference was observed between age groups—older people were relatively more interested in receiving advice regarding specific health solutions (ie, losing weight, avoiding illness, or improving specific health aspects), whereas young people were more holistically interested in improving their lifestyle (ie, feeling fitter, gaining more energy, or developing healthy eating habits) by means of personalized recommendations.

The applicability of the advice can be increased by the way in which the advice is formulated. As indicated previously, in the studies, the advice text contained practical tips (Textbox 2). Evaluation of the studies indicated that the provided personalized advice was perceived as helpful to improve diet, easy to understand, useful, and fun. More importantly, the groups of participants that received personalized advice better saw the link between their diet scores and showed greater improvement in dietary habits than the group of participants that did not receive personalized advice.

**Example of advice text along with practical tips on how to implement the advice (translated from the original Dutch text).**

**Example of advice text and practical tips**

- You eat few whole-grain products
- Because of your increased waist circumference, it is extra important for you to eat enough dietary fiber. Try to eat more products rich in dietary fiber. Tips for eating more dietary fiber:
  Try to choose whole meal bread more often and use whole meal products in the evening meal such as whole meal pasta, potatoes, and brown rice.
  Try to opt for legumes more often by adding lentils, chickpeas or kidney beans in a salad or soup, or eat a slice of whole meal bread with hummus.

### Source of Personalized Advice

The source of personalized advice influences perceived trustworthiness, relevance, and adherence. Previous studies show that a dietician is perceived as one of the most suitable providers of personalized nutrition advice [75]. In our study with *highly motivated consumers with premetabolic syndrome*, the advice was provided by a dietician, which was perceived as positive. However, advice obtained via telephone consultation was appreciated higher than that obtained via email. In consultation with the dietician, the advice generated by the model was discussed and further aligned to individual needs and capabilities, resulting in a behavior change strategy (eg, adjusting portion sizes or replacement of products within or outside the food category).

### Behavior Change Techniques

An important way to realize behavior change is through the application of behavior change techniques that are proven to be effective [76]. A behavior change technique is a strategy that helps an individual to change their behavior to promote better health. Michie et al [77] developed a hierarchically ordered taxonomy of 93 distinct behavior change techniques with labels, definitions, and examples. For our studies, we used one of these techniques, that is, implementation intentions, or if-then plans specifying when, where, and how one will achieve a certain behavior [70]. Previous research findings show that implementation intentions are more effective when consumers can formulate their own implementation intentions [78-80]. In addition, it is suggested that consumers can only handle a few behavior changes or implementation intentions at a time [78,81]. This could make implementation intentions a perfect tool to implement personalized advice. For example, in a pilot study among older consumers, participants were instructed to formulate implementation intentions in which they described how they planned to apply at least 2 pieces of the received personalized advice [24]. More specifically, participants had to explicitly indicate at which time of the day and in which situation they were planning to replace an unhealthy product that they reported in their 3-day food diary with a healthy product. However, as the results of this pilot study show that compliance with the personalized advice did not improve throughout the study period, replacing one food with a healthy alternative is likely to be insufficient. In our personalized advice system, we used free text to formulate the implementation intentions instead of using predefined text fields. In one study, a dietician helped consumers with formulating these implementation intentions, whereas in the other study, implementation intentions were formulated together with the researcher in the supermarket. Future studies have to determine how the effectiveness of implementation intentions can be further optimized and supported.

### Ethics Approval

The study with highly motivated consumers was conducted in accordance with the Declaration of Helsinki, and the protocol was approved by the ethics committee of Tilburg University (file number NL61382.028.17). For the study with consumers with low socioeconomic status, the ethics committee of Tilburg University (file number NW2017-42) determined that the study does not fall under the Medical Research Involving Human Subjects Act. All participants provided informed consent for inclusion before they participated in the studies.

## Discussion

### Summary

In this paper, we described the rationale behind the design choices made while developing our PNAS using the *sense, reason, and act* principle. This PNAS was tested in 2 studies, 1 with *highly motivated consumers with premetabolic syndrome* and 1 with *consumers with a low socioeconomic status*. The reasoning engine, a BBN, was identical for both studies and could handle different combinations of input parameters. For the study with highly motivated consumers, the resulting feedback and advice were displayed in a web-based portal. For the study with consumers with low socioeconomic status, feedback and advice were provided via an automatically generated report that was sent to the participants via email.

### Sensing

In terms of *sensing*, the main challenge is to decide which input parameters are relevant for your PNAS. The consumer burden of performing measurements should be weighed against their added value in providing a more personalized dietary recommendation. The main determinants in choosing measurement methods are their reliability, validity, responsiveness [82], and feasibility in a given setting (eg, do-it-yourself setting). Self-reporting can result in low data quality, irrespective of the method selected [83,84]. In contrast, the gold-standard method, such as using double-labeled water for caloric intake [85], is not always feasible in a real-life setting. The measurement method that is most suitable (eg, for measuring food intake) in a given setting depends on multiple factors, such as the required level of detail (food patterns, food groups, or specific nutrients), available guidance when collecting the data, and measurement frequency. When using self-reporting, it is important to design short questionnaires to obtain information from individuals. For example, Demark-Wahnefried et al [86] showed that intervention participants found brief interim surveys that assessed specific behaviors more helpful than long, standardized surveys. In addition, technology-based tools such as those using additional food images can help to improve the data accuracy of self-reported dietary assessments [87,88]. Finally, one may also consider adding supplementary sources, such as purchase data or dietary intake markers.

For health monitoring, new technologies and do-it-yourself options are becoming available. Health can be monitored via different types of wearables and at-home assessment kits (pregnancy test and genetic testing). However, the accuracy and validity of these measurement methods are not always guaranteed, especially in a do-it-yourself setting [89,90]. Collecting dried blood spots is possible in an at-home setting but requires high-quality blood spots and may be subject to undersampling or hematocrit bias or effect [91], and sufficient analysis capacity is required. The type of measurement that is acceptable also depends on the target group. Our studies showed that highly motivated individuals were more willing to accept blood sampling to receive more detailed personal advice [37] than individuals with low socioeconomic status who were not particularly interested in nutrition-related solutions for health [38].

The collected health data can also help to improve the algorithms underlying personalized advice. For instance, they can assist in identifying food-health relations at an individual level. The key is that the health information can be linked to nutritional recommendations. It can be argued that a single measurement is not sufficient and that long-term monitoring and reassessment are required to track changes in dietary behavior and health effects. With this input, the advice to the individual can be continuously updated and fine-tuned.

When providing feedback about health status, the potential for health gain for the individual should be clear. For instance, the Wii fit regularly shows consumers the difference between their real age and their *Wii fit age*. In theory, this could be a meaningful score, but in practice, it has been proven invalid and unreliable [92]. The new definition of health calls for alternative assessment methods that observe the body’s systemic response to variable circumstances instead of performing a single measurement, such as fasting glucose level [41]. This could be achieved by integrating multiple biomarkers or phenotypic traits into a single, understandable score that represents health [42]. In the future, other measurements could also be included (ie, mixed meal, exercise, or stress challenge tests [93]), but their added value for personalized nutrition and feasibility in practical conditions are still unclear.

In summary, regarding *sensing*, we recommend to try to find a good balance between the input parameters that you need to deliver the personalized dietary advice and the burden that you pose on your user—match the necessary parameters to the intended purpose of your PNAS and make a distinction between *must have* parameters and *nice to have* parameters. In addition, also in terms of repeated measurements, each time you ask a user for personal data, think about the value that you can provide them in return.

### Reasoning

Regarding *reasoning*, we discussed the use of a BBN for representing food-health relations. This approach allows combining a knowledge-driven approach with a data-driven approach; at first, a qualitative network can be created with nutritional experts, which can then be enhanced with observational data. However, the latter requires sufficiently large data sets. Therefore, in small studies, other knowledge-based modeling approaches may be more suitable, such as decision trees or system dynamics models [15]. The main challenge in such approaches is to determine the strength and reliability of the food-health relations. Ideally, we would assign a standardized *level of evidence* marker for each knowledge rule. With such a parameter, each user of a knowledge rule could decide which level of evidence is acceptable in a particular use case [50]. An advantage of expert-driven models is that they are transparent and can be based on biologically plausible mechanisms [94]. Advantages of a data-driven approach are that data from many different markers can be analyzed simultaneously, for instance, using machine learning techniques [13]. Disadvantages are that the interpretability of such models and algorithms may be low and that these methods are at risk of sampling and selection bias [17]. Hybrid models that combine both approaches could provide the best of both worlds. In any case, the developed models and PNAS systems as a whole should be validated using human intervention studies, published in peer-reviewed journals [17].

To summarize, for *reasoning*, the main message is to carefully consider whether a data-driven, knowledge-driven, or hybrid model is best suited for the intended PNAS. In addition, we recommend to not build your system around a particular technology or software solution. Technologies become obsolete, and it is risky to rely on a single software solution. Instead, think about the basic algorithm or knowledge rules that you aim for in your system and implement them in a modular architecture (ie, based on web services that can be maintained independently). In addition, ensure that you have a clear understanding of the role of the data that you build your system around and have a clear data security plan.

### Acting

For *acting*, we discussed the importance of proper communication of feedback and advice to consumers and the use of behavior change techniques for activating consumers (eg, implementation plans). Note that, until now, little to no scientific research has been conducted to determine how a personalized nutrition advice should best be communicated to consumers [95]. This is a serious shortcoming, as studies show that the way in which message content is processed and remembered greatly depends on how this information is delivered [96,97]. Relevant insights from social psychology and marketing literature can be useful to formulate advice for consumers that is effective in helping them to choose and maintain an optimal personalized diet.

In developing our PNAS, we also considered the format of the feedback and advice provided. For example, to make feedback about health status more easily understandable, color coding or composite scores were used. At this point, composite scores mostly represent a specific aspect of health, such as metabolic health, muscle health, or inflammation status [24,98]. The integration of all markers in a single health score could be even more effective. Other approaches that integrate multiple health aspects in a single visualization are already available, but none of these provide a single health score [73,99]. The influence of composite health scores on consumer understanding, motivation, and behavior change is not sufficiently known yet.

The advice we presented in the studies contained practical tips, making the advice more easily applicable in daily life. Ideally, the framing and format of such advice is adjusted to personal characteristics of the receiver of the advice. In one of our studies, this was accomplished by a dietician, who adapted the advice to a practical application for the participant. However, ideally, a digital advice system also takes these personal needs into account. This may involve tailoring the framing and timing of the advice to specific personality types [100]. Previous studies have shown that both an entirely digital approach and a digital approach combined with coaching resulted in healthy dietary behavior; however, user engagement was higher in the combined approach [101].

In addition, we asked participants to formulate implementation intentions. Future studies should examine other behavior change techniques also. As an extended list of techniques exist (eg, as specified by Michie et al [76]) that could potentially add to the effectiveness of personalized dietary advice, we recommend future studies to incorporate a broad range of behavior change techniques and identify potential synergies by combining different behavior change techniques. For example, Social Cognitive Theory [102] provides insight into how individuals regulate their behavior to achieve goals that can be maintained over time. For example, self-efficacy, defined as the extent to which one believes in their own ability to reach a certain goal, has an effect on actually reaching that goal [102]. However, self-efficacy is difficult to influence, also when using personalized advice, as shown by Doets et al [24]. They found that self-efficacy decreased during the study in both the intervention group that received personalized nutrition advice and the control group that received generic dietary advice. This reduced self-efficacy could also be a result of the fact that during the study, participants were confronted with their (unhealthy) diet, which may lead to lack of confidence in someone’s ability to succeed [103]. In their meta-analysis, Prestwich et al [104] revealed that emotional stress could undermine a positive effect on self-efficacy. Interventions that incorporate techniques that help to manage this stress were more successful in raising dietary self-efficacy than interventions that do not. Therefore, we recommend future studies in the context of personalized nutrition advice to also include some type of stress management technique as part of the intervention.

To summarize, regarding *acting*, it is important to tailor the communication of the advice to the target group, in terms of format, choice of words, and complexity. Moreover, use different behavior change techniques and choose the technique that best meets the needs of the target group.

### Conclusions

To set up or further develop a personalized advice system considering a sense, reason, and act approach is of value. It provides guidance and structure for making design choices for developing a (partly) digitalized solution. Besides the type of personal dietary advice and the mode through which it is generated, the communication of the advice and appropriate behavior change techniques should be considered, as this will determine the level of consumer adherence. This paper shows the vastness of choice options in designing personalized advice systems. All these choices will eventually influence the functionality, complexity, consumer appreciation, and effectiveness in achieving the desired health outcomes of such systems.

## Appendix

Multimedia Appendix 1 Example feedback report about dietary intake status, as provided in the study with consumers with low socioeconomic status. The number of stars for a specific food group indicates the level of compliance with the Dutch dietary guidelines.

## Abbreviations

BBN: Bayesian Belief Network
DHEI: Dutch Healthy Eating Index
HDL: high-density lipoprotein
LDL: low-density lipoprotein
PNAS: personalized nutrition advice system
