# Supplementary material for: Design Issues in Personalized Nutrition Advice Systems
Source: J Med Internet Res. 2023 Mar 29;25:e37667. doi: 10.2196/37667 (PMC10131983; doi:10.2196/37667)

Scores on the Healthy Eating Questionnaire

Here you can read for 9 categories how well you score in terms of healthy eating. The number of stars per category indicates how well you are doing. The more stars, the better. The category with most room for improvement is listed on top.

Choose one category for which you would like to get personalized advice. You can view a maximum of 5 advices. It is most valuable if you choose a category for which you score few stars.

Unhealthy choices

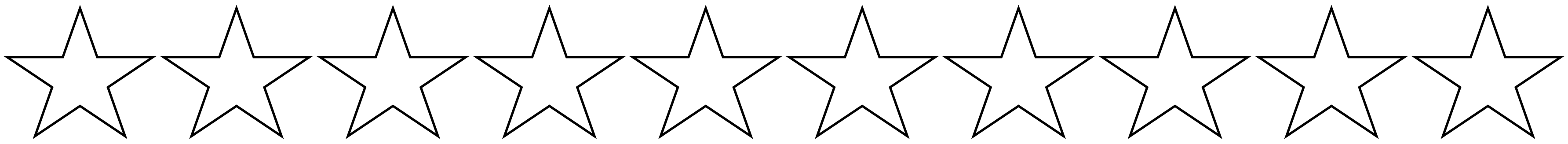

Dairy

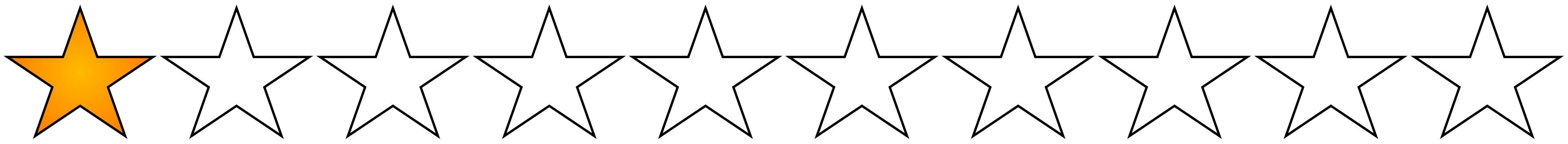

Salt

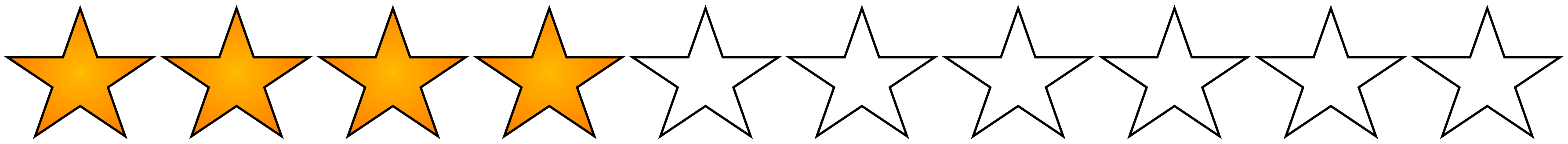

Sugar sweetened beverages

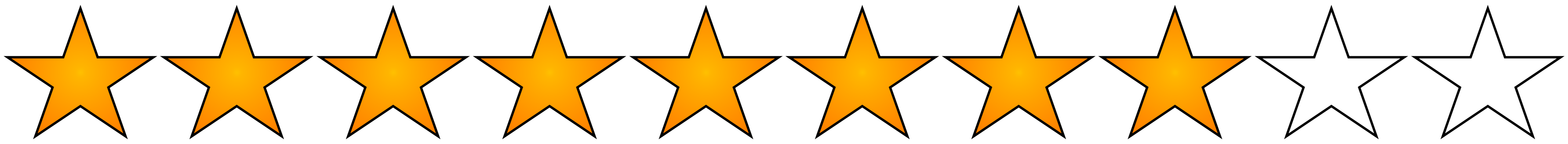

Vegetables

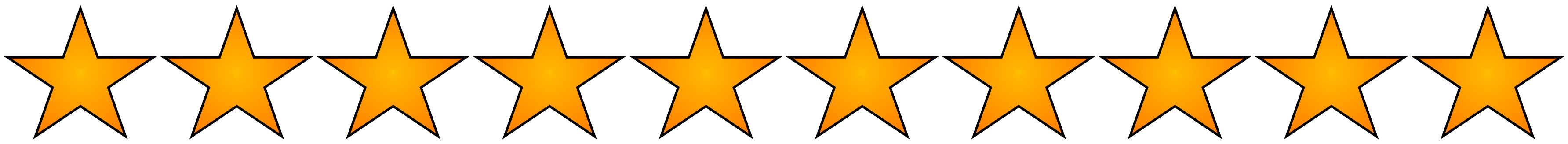

Supplement: Multimedia Appendix 1 [file jmir_v25i1e37667_app1.pdf]
